# Maternal Delivery Outcomes Associated With Acute Sickle Cell Crisis: A National Inpatient Analysis

**DOI:** 10.7759/cureus.116136

**Published:** 2026-09-12

**Authors:** John R Passman, Danielle Fastring

**Affiliations:** 1 Medicine, William Carey University College of Osteopathic Medicine, Hattiesburg, USA; 2 Research and Preclinical Sciences, William Carey University College of Osteopathic Medicine, Hattiesburg, USA

**Keywords:** cesarean delivery (cd), hemolysis elevated liver enzymes and low platelets (hellp), hypertensive disorders, national inpatient sample (nis), preeclampsia, pregnancy, sickle cell crisis, sickle cell disease (scd)

## Abstract

Background: Pregnancies complicated by sickle cell disease (SCD) carry substantial maternal and perinatal risk, but the specific association between acute sickle cell crisis (SCC) and adverse obstetric outcomes at delivery remains incompletely characterized. This study evaluated whether acute sickle cell crisis was associated with hypertensive disorders of pregnancy and cesarean delivery among delivery hospitalizations involving SCD.

Methods: We performed a retrospective cross-sectional analysis of the 2021 Healthcare Cost and Utilization Project National Inpatient Sample (HCUP-NIS). Delivery hospitalizations involving SCD were identified using the International Classification of Diseases, Tenth Revision, Clinical Modification (ICD-10-CM) codes and classified as involving acute sickle cell crisis or SCD without crisis. The primary outcome was a composite of severe preeclampsia, eclampsia, or hemolysis, elevated liver enzymes, and low platelets (HELLP) syndrome. Secondary outcomes were any preeclampsia, eclampsia, or HELLP syndrome and cesarean delivery. Survey-weighted logistic regression estimated crude and adjusted odds ratios while accounting for the complex sampling design. Adjusted models included maternal age, Black versus non-Black race and ethnicity, and obesity.

Results: The final cohort included 508 unweighted hospitalizations, representing an estimated 2,540 delivery hospitalizations nationally; 68 (13.4%) involved acute crisis, and 440 (86.6%) involved SCD without crisis. Percentages for study outcomes are survey-weighted, and n values are unweighted. Severe preeclampsia, eclampsia, or HELLP syndrome occurred in 19 (27.9%) hospitalizations involving acute crisis compared with 30 (6.8%) without crisis. Acute crisis was associated with higher adjusted odds of the primary outcome (adjusted odds ratio (aOR): 4.63; 95% confidence interval (CI): 2.43-8.85; p<0.001). The association remained significant after additional adjustment for gestational age (aOR: 4.27; 95% CI: 2.11-8.62; p<0.001). Acute crisis was also associated with any preeclampsia, eclampsia, or HELLP syndrome (aOR: 3.62; 95% CI: 1.96-6.71; p<0.001) and cesarean delivery (aOR: 2.18; 95% CI: 1.30-3.68; p=0.003).

Conclusion: Among delivery hospitalizations involving SCD, acute sickle cell crisis was strongly associated with severe hypertensive disorders of pregnancy and cesarean delivery. Acute crisis may identify a particularly high-risk clinical state during delivery hospitalization and may warrant close surveillance and coordinated multidisciplinary management.

## Introduction

Sickle cell disease (SCD) is one of the most commonly inherited genetic conditions worldwide, characterized by chronic hemolytic anemia, vaso-occlusive episodes, and progressive organ injury [1]. With more than 500,000 infants born with the condition annually, the global burden is substantial and requires specialized obstetric management [2,3]. Advances in medical care have enhanced survival rates for those with SCD, allowing a greater number of individuals with SCD to reach reproductive age [4,5]. Consequently, pregnancies complicated by SCD are becoming more common and are consistently described as high-risk for severe maternal and perinatal adverse events [6].

Physiologically, pregnancy imposes distinct demands on patients with SCD. SCD is associated with chronic inflammation, endothelial dysfunction, and vascular injury resulting from interactions among sickled erythrocytes, leukocytes, platelets, and the vascular endothelium. These interactions trigger the release of proinflammatory cytokines and contribute to vascular dysfunction [7]. The normal vascular and inflammatory adaptations of pregnancy may aggravate these pathophysiological alterations, thereby increasing susceptibility to microvascular injury and ensuing complications for both the pregnant patient and fetus [6]. A sickle cell crisis (SCC) occurs when sickled red blood cells adhere to blood vessel walls, slowing or blocking blood flow in small vessels, leading to reduced oxygen delivery and tissue ischemia. This process represents a central mechanism underlying many SCD-related complications [8]. Vaso-occlusive crises are more common during pregnancy and represent one of the most frequent maternal complications among pregnant patients with sickle hemoglobinopathies [9,10].

Pregnancy outcomes for patients with SCD have not significantly improved over time, and the frequency of certain complications has increased. Women with SCD continue to face a higher risk of severe morbidity relative to the general population, with maternal mortality rates persisting near 1 per 200 pregnancies [11]. A 2015 systematic review and meta-analysis including 16 studies across 9 countries identified an increased risk of intrauterine growth restriction (IUGR) and perinatal mortality among infants born to patients with SCD. These risks remained consistent across both low-income and high-income countries [12].

Recent analyses of large administrative datasets have demonstrated that pregnant patients with SCD experience substantially higher rates of severe maternal morbidity, maternal mortality, and adverse obstetric outcomes than women without SCD [11,13,14]. These studies have leveraged nationally representative datasets such as the National Inpatient Sample (NIS) to characterize pregnancy outcomes in this high-risk population. However, less is known regarding the specific association between acute sickle cell crisis and hypertensive disorders of pregnancy or cesarean delivery among delivery hospitalizations involving SCD. Acute crisis may identify a subgroup experiencing greater vascular, inflammatory, and ischemic stress during delivery hospitalization. Therefore, the primary purpose of this study was to evaluate the association between acute sickle cell crisis and a composite of severe preeclampsia, eclampsia, or hemolysis, elevated liver enzymes, and low platelets (HELLP) syndrome among delivery hospitalizations involving SCD. Secondary objectives were to evaluate associations with a broader composite of preeclampsia, eclampsia, or HELLP syndrome and with cesarean delivery. We hypothesized that delivery hospitalizations involving acute sickle cell crisis would have higher odds of severe preeclampsia, eclampsia, or HELLP syndrome and cesarean delivery than delivery hospitalizations involving SCD without crisis.

## Materials and methods

Data from the 2021 Healthcare Cost and Utilization Project National Inpatient Sample (HCUP-NIS) were analyzed in this retrospective cross-sectional study. The NIS is a stratified sample of discharges from United States community hospitals and provides discharge-level weights that permit calculation of national estimates. Analyses incorporated the NIS discharge weight (DISCWT), hospital stratum (NIS_STRATUM), and hospital cluster identifier (HOSP_NIS) to account for the complex sampling design.

Delivery hospitalizations were identified using HCUP’s International Classification of Diseases, Tenth Revision, Clinical Modification (ICD-10-CM) delivery indicator. We then restricted the cohort to delivery hospitalizations with a diagnosis of sickle cell disease recorded in any of the 40 diagnosis fields available in the NIS. Hospitalizations assigned to Medicare Severity Diagnosis-Related Groups (MS-DRGs) 783, 784, 785, 796, 797, or 798 were excluded because these DRGs include sterilization or dilation and curettage procedures that could affect classification of operative delivery. The study cohort selection process is summarized in Figure 1.

**Figure 1:**
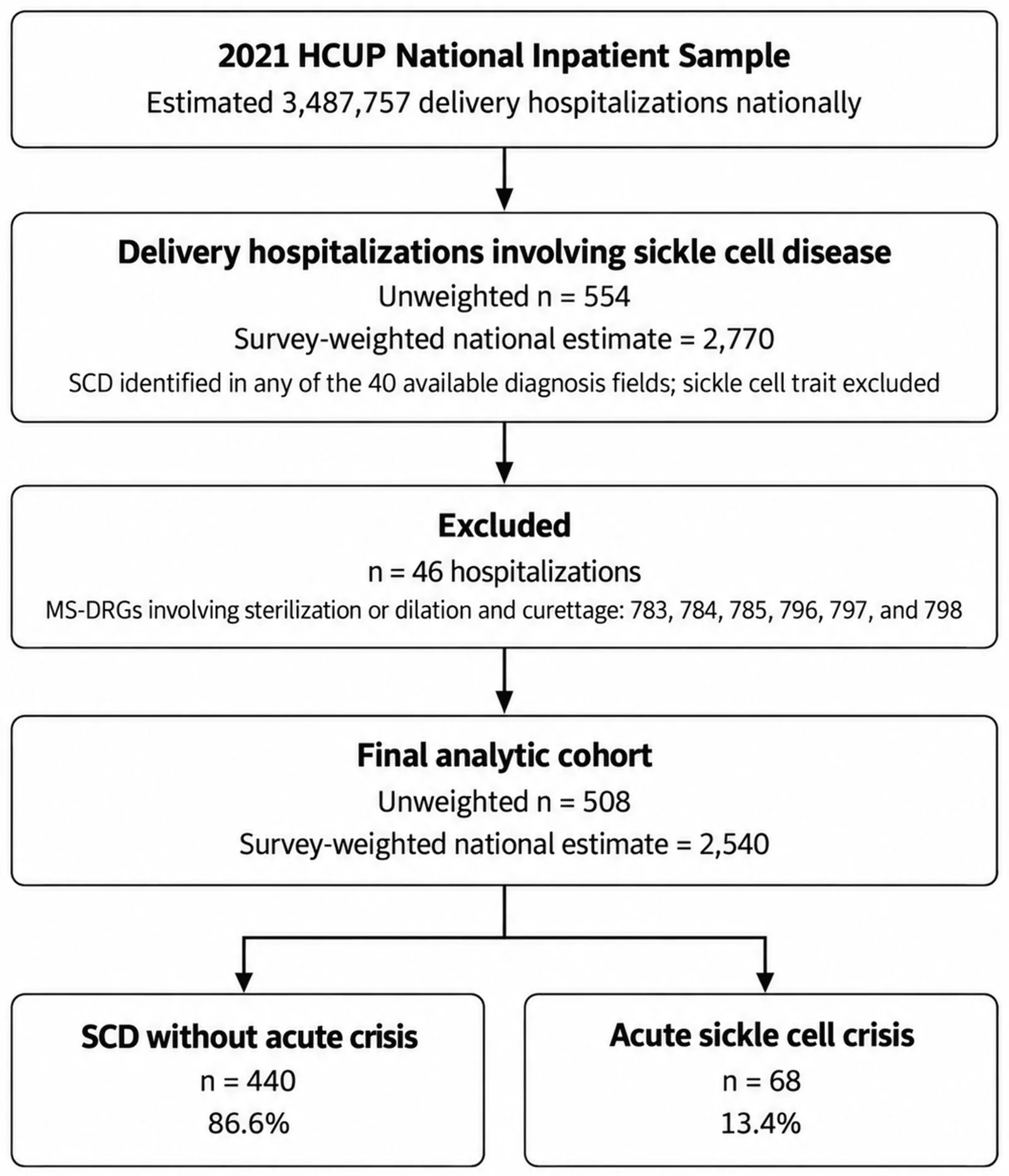
Study cohort selection Figure note: Delivery hospitalizations involving sickle cell disease were identified from the 2021 Healthcare Cost and Utilization Project National Inpatient Sample. Of 554 unweighted SCD delivery hospitalizations, 46 hospitalizations assigned to prespecified MS-DRGs involving sterilization or dilation and curettage were excluded, leaving 508 hospitalizations in the final analytic cohort. Of these, 440 were classified as SCD without acute crisis and 68 as acute sickle cell crisis. National estimates were calculated using NIS discharge weights. HCUP: Healthcare Cost and Utilization Project, MS-DRG: Medicare Severity Diagnosis-Related Group, NIS: National Inpatient Sample, SCD: sickle cell disease

Sickle cell disease was identified using ICD-10-CM diagnosis codes for Hb-SS disease, sickle cell/Hb-C disease, sickle cell thalassemia, and other sickle cell disorders. Sickle cell trait was excluded. The exposure of interest was acute sickle cell crisis. Hospitalizations were classified as involving acute crisis when any diagnosis field contained an ICD-10-CM code indicating sickle cell disease with crisis, including unspecified crisis, acute chest syndrome, splenic sequestration, cerebral vascular involvement, dactylitis, or another specified crisis. Hospitalizations containing a sickle cell disease code without crisis and no code indicating crisis were classified as sickle cell disease without crisis. When both crisis and non-crisis codes were present, the hospitalization was classified as involving acute crisis. The complete list of ICD-10-CM codes is provided in Table 1.

**Table 1:** Diagnosis and procedure definitions used in the analysis Table note: ICD-10-CM diagnosis codes were evaluated across all 40 diagnosis fields, and ICD-10-PCS cesarean codes were evaluated across all available procedure fields. Sickle cell trait, D57.3, was excluded. When both crisis and non-crisis SCD codes were present, the hospitalization was classified as involving acute crisis. Codes are displayed with decimal points for readability; NIS diagnosis fields store ICD-10-CM codes without decimals. The complete SCD definition includes the reviewer-questioned codes D57.219, D57.419, and D57.819. The SCD code families and cesarean procedure definitions are consistent with CMS code descriptions. HCUP: Healthcare Cost and Utilization Project, HELLP: hemolysis, elevated liver enzymes, and low platelets, ICD-10-CM: International Classification of Diseases, Tenth Revision, Clinical Modification, ICD-10-PCS: International Classification of Diseases, Tenth Revision, Procedure Coding System, MS-DRG: Medicare Severity Diagnosis-Related Group, NIS: National Inpatient Sample, SCD: sickle cell disease

Study variable	ICD-10-CM, ICD-10-PCS, or HCUP definition	Operational definition
Sickle cell disease without acute crisis	D57.1; D57.20; D57.40; D57.42; D57.44; D57.80	Delivery hospitalization containing a sickle cell disease diagnosis explicitly designated as without crisis
Hb-SS disease with acute crisis	D57.00; D57.01; D57.02; D57.03; D57.04; D57.09	Hb-SS disease with unspecified crisis, acute chest syndrome, splenic sequestration, cerebral vascular involvement, dactylitis, or another specified crisis complication
Sickle cell/Hb-C disease with acute crisis	D57.211; D57.212; D57.213; D57.214; D57.218; D57.219	Sickle cell/Hb-C disease with acute chest syndrome, splenic sequestration, cerebral vascular involvement, dactylitis, another specified crisis complication, or unspecified crisis
Sickle cell thalassemia, unspecified, with acute crisis	D57.411; D57.412; D57.413; D57.414; D57.418; D57.419	Sickle cell thalassemia, unspecified type, with acute chest syndrome, splenic sequestration, cerebral vascular involvement, dactylitis, another specified crisis complication, or unspecified crisis
Sickle cell thalassemia beta-zero with acute crisis	D57.431; D57.432; D57.433; D57.434; D57.438; D57.439	Sickle cell thalassemia beta-zero with acute chest syndrome, splenic sequestration, cerebral vascular involvement, dactylitis, another specified crisis complication, or unspecified crisis
Sickle cell thalassemia beta-plus with acute crisis	D57.451; D57.452; D57.453; D57.454; D57.458; D57.459	Sickle cell thalassemia beta-plus with acute chest syndrome, splenic sequestration, cerebral vascular involvement, dactylitis, another specified crisis complication, or unspecified crisis
Other sickle cell disorders with acute crisis	D57.811; D57.812; D57.813; D57.814; D57.818; D57.819	Other sickle cell disorder with acute chest syndrome, splenic sequestration, cerebral vascular involvement, dactylitis, another specified crisis complication, or unspecified crisis
Mild to moderate preeclampsia	O14.00; O14.02; O14.03; O14.04; O14.05	Mild to moderate preeclampsia during an unspecified trimester, second trimester, third trimester, childbirth, or puerperium
Severe preeclampsia	O14.10; O14.12; O14.13; O14.14; O14.15	Severe preeclampsia during an unspecified trimester, second trimester, third trimester, childbirth, or puerperium
HELLP syndrome	O14.20; O14.22; O14.23; O14.24; O14.25	Hemolysis, elevated liver enzymes, and low platelet count syndrome during an unspecified trimester, second trimester, third trimester, childbirth, or puerperium
Eclampsia	O15.0; O15.1; O15.2; O15.9	Eclampsia occurring during pregnancy, labor, the puerperium, or an unspecified time period
Severe hypertensive disorder composite	Any severe preeclampsia, HELLP syndrome, or eclampsia code listed above	Primary outcome
Any preeclampsia, eclampsia, or HELLP syndrome	Any mild-to-moderate preeclampsia, severe preeclampsia, HELLP syndrome, or eclampsia code listed above	Secondary outcome
Cesarean delivery	10D00Z0; 10D00Z1; 10D00Z2	Extraction of products of conception by an open approach, including high/classical, low cervical, and extraperitoneal cesarean delivery
Delivery hospitalization	I10_DELIVERY = 1	HCUP ICD-10-CM delivery indicator
Excluded MS-DRGs	783; 784; 785; 796; 797; 798	Delivery hospitalizations involving sterilization or dilation and curettage were excluded from the final analytic cohort
Obesity	E66.x	Presence of an obesity diagnosis in any diagnosis field
Type 2 diabetes	E11.x	Presence of a type 2 diabetes diagnosis in any diagnosis field
Chronic kidney disease	N18.x	Presence of a chronic kidney disease diagnosis in any diagnosis field
Multiple gestation	O30.x	Presence of a multiple gestation diagnosis in any diagnosis field

The primary outcome was a composite of severe preeclampsia, eclampsia, or HELLP syndrome identified from ICD-10-CM diagnosis codes recorded in any diagnosis field. Secondary outcomes were a broader composite of any preeclampsia, eclampsia, or HELLP syndrome and cesarean delivery. The broader hypertensive disorder composite included mild-to-moderate preeclampsia, severe preeclampsia, HELLP syndrome, and eclampsia. Cesarean delivery was identified using prespecified International Classification of Diseases, Tenth Revision, Procedure Coding System (ICD-10-PCS) procedure codes recorded in any available procedure field. Outcome definitions and codes are provided in Table 1. Because the NIS is an administrative discharge database and does not contain the complete clinical measurements required to independently adjudicate hypertensive disorders of pregnancy, preeclampsia, eclampsia, and HELLP syndrome were operationally defined by the corresponding prespecified ICD-10-CM diagnosis codes listed in Table 1 rather than by independently applying clinical diagnostic criteria based on blood pressure, laboratory, or other clinical data.

Covariates were selected a priori based on prior literature and clinical relevance as potential confounders of the association between acute sickle cell crisis and the study outcomes. The primary adjusted models included maternal age at admission, race and ethnicity, and obesity. Maternal age was modeled continuously. Because of small cell sizes, race and ethnicity were categorized as Black versus non-Black for multivariable analyses. Race and ethnicity were analyzed as reported in the NIS and were not inferred from sickle cell disease status. Obesity was defined using prespecified ICD-10-CM diagnosis codes. Type 2 diabetes mellitus, chronic kidney disease, and multiple gestation were described but were not included in adjusted models because of extreme sparsity within the analytic cohort. Gestational age was not included in the primary model because it could occur downstream of both acute crisis and severe hypertensive disease. It was included in a sensitivity analysis.

Multivariable models used complete-case analysis for the variables included in each model. Missing race and ethnicity values were not imputed or modeled as a separate category.

Categorical variables were summarized using unweighted frequencies and survey-weighted percentages. Continuous variables were summarized using survey-weighted means and design-adjusted standard errors. National estimates were calculated using DISCWT, and variance estimation accounted for stratification by NIS_STRATUM and clustering by HOSP_NIS. Survey-weighted logistic regression was used to estimate crude and adjusted odds ratios (aORs) with 95% confidence intervals (CIs) for the association between acute sickle cell crisis and each outcome. Adjusted models included maternal age, Black versus non-Black race and ethnicity, and obesity. All prespecified covariates were entered simultaneously into each multivariable logistic regression model in a single block and were retained regardless of statistical significance. The adjusted severe hypertensive disorder, broader hypertensive disorder, and cesarean delivery models each included 492 complete cases. Gestational age was derived from ICD-10-CM Z codes indicating completed weeks of gestation and was added to the same covariate set only in the sensitivity analysis of the primary outcome; this sensitivity model included 476 complete cases. The six-category NIS race/ethnicity variable was collapsed to Black versus non-Black for multivariable analyses, with all other non-missing categories assigned to the non-Black group and missing values retained as missing. Analyses were conducted using IBM SPSS Statistics version 30 (IBM Corp., Armonk, NY). Complex Samples analyses incorporated DISCWT as the sampling weight, NIS_STRATUM as the stratification variable, HOSP_NIS as the cluster variable, and the final analytic cohort as the analysis domain. Automated stepwise selection and univariate p-value screening were not used. Multicollinearity among model covariates was assessed using tolerance and variance inflation factors (VIFs), with tolerance values >0.10 and VIFs <10 considered indicative of acceptable collinearity. Continuous predictors were evaluated for plausibility and appropriate ranges before analysis. Frequency distributions and range checks were used to identify missing, implausible, or inconsistent values. One implausible gestational age value of one week was recoded as missing. Because the NIS is an administrative discharge database, diagnoses and procedures could not be independently validated against source medical records. The sensitivity analysis repeated the severe hypertensive disorder model with additional adjustment for gestational age. Statistical tests were two-sided, and p values less than 0.05 were considered statistically significant. Analyses were conducted using IBM SPSS Statistics Complex Samples procedures.

The study was conducted in accordance with applicable United States Department of Health and Human Services regulations at 45 CFR 46, as well as the HCUP Data Use Agreement. Because this study involved only deidentified secondary data and no interaction or intervention with human participants, the provisions of the Declaration of Helsinki related to enrollment and informed consent were not directly applicable. Nevertheless, the study was conducted in accordance with applicable ethical principles governing confidentiality, privacy protection, and responsible secondary use of health data.

## Results

Among an estimated 3,487,757 delivery hospitalizations in the United States in 2021, an estimated 2,770 (0.079%) involved sickle cell disease, equivalent to a prevalence of 7.94 per 10,000 deliveries. The unweighted SCD delivery cohort included 554 hospitalizations. After excluding 46 hospitalizations assigned to MS-DRGs involving sterilization or dilation and curettage, the final analytic cohort included 508 unweighted hospitalizations, representing an estimated 2,540 hospitalizations nationally. Of these, 68 (13.4%) hospitalizations involved acute sickle cell crisis and 440 (86.6%) involved sickle cell disease without crisis. Race and ethnicity were missing for 16 (3.1%) hospitalizations. Gestational age was also missing for 16 (3.1%) hospitalizations.

The survey-weighted mean maternal age in the final analytic cohort was 27.9 years. Among hospitalizations without crisis, 334 (78.6%) were among Black patients, and 56 (12.7%) included an obesity diagnosis. Among hospitalizations with acute crisis, 64 of 67 (95.5%) were among Black patients, and 5 (7.4%) included an obesity diagnosis. Race and ethnicity percentages were calculated among hospitalizations with non-missing race and ethnicity data. Maternal and obstetric characteristics stratified by acute sickle cell crisis status are presented in Table 2.

**Table 2:** Characteristics of delivery hospitalizations involving sickle cell disease, stratified by acute crisis status Table note: Values are unweighted frequencies with survey-weighted percentages unless otherwise indicated. Means and percentages incorporate the NIS discharge weight, and standard errors account for NIS stratification and hospital clustering. Race and ethnicity data were available for 425 hospitalizations without crisis and 67 with crisis. Gestational age data were analyzed after one implausible value of one week was recoded as missing. Statistical tests used to generate the reported p values were as follows: maternal age and gestational age were evaluated using design-adjusted Complex Samples general linear models; Black race and ethnicity and obesity were evaluated using unadjusted Complex Samples logistic regression; and type 2 diabetes, chronic kidney disease, and multiple gestation were evaluated using Fisher’s exact tests because of sparse cell counts. NIS: National Inpatient Sample, SE: standard error

Characteristic	No crisis, n=440	Acute crisis, n=68	p value
Maternal age, weighted mean±SE, years	27.97±0.28	27.29±0.74	0.396
Black race and ethnicity, number/available number (weighted %)	334/425 (78.6%)	64/67 (95.5%)	0.004
Obesity, number (weighted %)	56 (12.7%)	5 (7.4%)	0.193
Type 2 diabetes, number (%)	2 (0.5%)	1 (1.5%)	0.351
Chronic kidney disease, number (%)	2 (0.5%)	0 (0.0%)	1.000
Multiple gestation, number (%)	14 (3.2%)	0 (0.0%)	0.234
Gestational age, weighted mean±SE, weeks	37.55±0.17	35.30±0.44	<0.001

Severe preeclampsia, eclampsia, or HELLP syndrome occurred in 19 (27.9%) hospitalizations involving acute sickle cell crisis and 30 (6.8%) hospitalizations without crisis. In survey-weighted unadjusted analysis, acute crisis was associated with higher odds of severe hypertensive outcome (OR: 5.29; 95% CI: 2.82-10.00; p<0.001). After adjustment for maternal age, Black versus non-Black race and ethnicity, and obesity, acute crisis remained associated with higher odds of severe preeclampsia, eclampsia, or HELLP syndrome (aOR: 4.63; 95% CI: 2.43-8.85; p<0.001). In a sensitivity model additionally adjusting for gestational age, acute sickle cell crisis remained associated with severe preeclampsia, eclampsia, or HELLP syndrome (aOR: 4.27; 95% CI: 2.11-8.62; p<0.001).

Any preeclampsia, eclampsia, or HELLP syndrome occurred in 24 (35.3%) hospitalizations with acute crisis and 53 (12.0%) hospitalizations without crisis. Acute crisis was associated with higher unadjusted odds of the broader hypertensive disorder composite (OR: 3.98; 95% CI: 2.16-7.30; p<.001), and the association remained significant after adjustment for maternal age, Black versus non-Black race and ethnicity, and obesity (aOR: 3.62; 95% CI: 1.96-6.71; p<0.001).

Cesarean delivery occurred in 43 (63.2%) hospitalizations involving acute crisis and 195 (44.3%) hospitalizations without crisis. Acute crisis was associated with higher unadjusted odds of cesarean delivery (OR: 2.16; 95% CI: 1.29-3.62; p=0.004), and the association remained after adjustment (aOR: 2.18; 95% CI: 1.30-3.68; p=0.003). The crude and adjusted associations between acute sickle cell crisis and the maternal outcomes are summarized in Table 3.

**Table 3:** Association of acute sickle cell crisis with maternal outcomes Table note: Values are unweighted frequencies with survey-weighted percentages. Crude and adjusted OR account for the NIS discharge weight, stratification, and hospital clustering. Adjusted models included maternal age, Black versus non-Black race and ethnicity, and obesity. P values were obtained from survey-weighted Complex Samples logistic regression models. Complete-case analysis was used for variables included in each model. OR compare delivery hospitalizations involving acute sickle cell crisis with those involving sickle cell disease without crisis. CI: confidence interval, HELLP: hemolysis, elevated liver enzymes, and low platelet count, NIS: National Inpatient Sample, OR: odds ratio

Outcome	No crisis, weighted %	Acute crisis, weighted %	Crude OR (95% CI)	Adjusted OR (95% CI)	Adjusted p value
Severe preeclampsia, eclampsia, or HELLP syndrome	30 (6.8%)	19 (27.9%)	5.29 (2.82-10.00)	4.63 (2.43-8.85)	<0.001
Any preeclampsia, eclampsia, or HELLP syndrome	53 (12.0%)	24 (35.3%)	3.98 (2.16-7.30)	3.62 (1.96-6.71)	<0.001
Cesarean delivery	195 (44.3%)	43 (63.2%)	2.16 (1.29-3.62)	2.18 (1.30-3.68)	0.003

## Discussion

Advances in early diagnosis and pediatric care have allowed more individuals with SCD to survive to reproductive age, increasing the need for specialized obstetric care for this high-risk population [3,15]. Although a multidisciplinary care approach has improved outcomes over time, pregnancy in patients with SCD continues to carry substantial maternal and fetal risk. One study in 2024 showed a maternal mortality rate of 1.6 per 1,000 births, illustrating that pregnancy alongside SCD still presents an increased risk of mortality despite improvements in care [10,13]. Additional data demonstrate elevated rates of maternal and fetal complications and indicate that substantial pregnancy-related risk persists in this population [10,16].

In this nationally representative analysis of delivery hospitalizations involving SCD, acute SCC was strongly associated with markedly higher odds of severe preeclampsia, eclampsia, or HELLP syndrome. The severe hypertensive disorder composite occurred in 27.9% of hospitalizations involving acute crisis compared with 6.8% of those without crisis. After adjustment for maternal age, Black versus non-Black race and ethnicity, and obesity, acute crisis was associated with more than fourfold higher odds of the primary outcome. Acute crisis was also associated with higher odds of the broader hypertensive disorder composite and cesarean delivery. These findings suggest that delivery hospitalizations involving acute crisis represent a particularly high-risk clinical state among patients who already have SCD.

Prior studies have shown that pregnant patients with SCD experience higher rates of maternal morbidity than the general obstetric population [17]. Fewer analyses have isolated acute SCC as a distinct exposure at the time of delivery [17,18]. Our findings extend this literature by demonstrating an association between acute crisis and clinically important hypertensive disorders of pregnancy among delivery hospitalizations involving SCD. This interpretation is consistent with prior work that examined data from 1999 to 2008 and found that SCC at the time of delivery was associated with increased labor and delivery complications [13]. Together, these findings support viewing acute SCC as a particularly high-risk clinical state rather than treating all SCD diagnoses as equivalent.

Several pathophysiological mechanisms may contribute to the observed association between acute crisis and hypertensive disorders of pregnancy. SCD is characterized by chronic hemolysis, inflammation, endothelial dysfunction, vascular injury, and episodic vaso-occlusion. During an acute crisis, worsening vascular obstruction, ischemia, and inflammatory activation may compound the endothelial and vascular changes associated with preeclampsia and related disorders. However, the cross-sectional design and discharge-level data do not establish whether acute crisis preceded the hypertensive disorder, resulted from the physiologic stress of the complication, or occurred concurrently. Therefore, the findings should be interpreted as associations rather than evidence that crisis directly caused the outcomes.

The broader composite of any preeclampsia, eclampsia, or HELLP syndrome was identified in 35.3% of hospitalizations involving acute crisis compared with 12.0% of hospitalizations without crisis. The persistence of the association after adjustment suggests that the finding was not explained solely by differences in maternal age, race and ethnicity, or obesity between the groups. Nevertheless, residual confounding remains possible because the NIS does not contain detailed measures of baseline SCD severity, frequency of prior crises, prenatal care, medication use, laboratory findings, or other clinical characteristics that may influence both crisis occurrence and hypertensive risk.

Acute crisis was also associated with approximately twofold higher adjusted odds of cesarean delivery. This association may reflect greater maternal or fetal acuity, concern for clinical deterioration, or a greater likelihood of medically indicated operative delivery among patients experiencing acute crisis. The NIS does not provide sufficient clinical detail to determine the specific indication for cesarean delivery or whether crisis began before the decision to perform the procedure. Consequently, the cesarean finding should not be interpreted as demonstrating that acute crisis itself necessitated operative delivery.

Gestational age was lower among hospitalizations involving acute crisis than among those without crisis. Prior studies have demonstrated that patients with SCD are more likely to deliver preterm than patients without SCD [19]. In the sensitivity analysis, acute crisis remained associated with severe preeclampsia, eclampsia, or HELLP syndrome after additional adjustment for gestational age, although the estimate was modestly attenuated. This finding supports the robustness of the primary association. However, gestational age was excluded from the primary model because earlier delivery may occur downstream of acute crisis, severe hypertensive disease, or both. Adjusting for a variable on the potential causal pathway could introduce overadjustment or collider bias. The gestational age model should therefore be considered a sensitivity analysis rather than the principal adjusted estimate.

Most delivery hospitalizations involving SCD occurred among patients categorized as Black, and the proportion was higher in the acute crisis group than in the group without crisis. This finding reflects the epidemiology of SCD in the United States and should not be interpreted as evidence that race itself is a biological cause of crisis or hypertensive complications. National studies have documented persistent racial inequities in severe maternal morbidity and mortality, including among Black patients [20,21]. SCD may contribute to these disparities because its burden falls disproportionately on Black communities, while structural inequities in access, continuity, quality of care, and social conditions may further influence maternal outcomes [6,14,22,23].

This study has several strengths. The use of a large, nationally representative sample of US delivery hospitalizations allowed us to examine a relatively uncommon but important exposure, acute SCC, in relation to severe hypertensive disorders of pregnancy and cesarean delivery. The complex sampling design and discharge weights permitted national estimates while accounting for hospital clustering and stratification. The size and scope of the HCUP-NIS also improve the generalizability of our findings across inpatient delivery settings in the United States. In addition, this study distinguished acute SCC from SCD more broadly, allowing us to evaluate whether crisis at the time of delivery represents a specific high-risk state. The exposure definition included the complete range of applicable crisis codes, and covariates were selected a priori rather than through automated selection or univariate statistical screening.

Several limitations should also be considered. As with any administrative database study, our findings depend on the accuracy of ICD-10-CM diagnosis and ICD-10-PCS procedure coding and are therefore subject to misclassification of both exposure and outcomes. Acute crisis may have been undercoded, and diagnosis fields do not reliably establish the timing or clinical severity of the crisis or hypertensive disorder. Because diagnoses and procedures could not be independently validated against clinical records, residual misclassification bias cannot be excluded. We also lacked detailed clinical information, including disease severity, laboratory values, medication use, and details of intrapartum management, all of which may influence maternal risk. The database does not provide information on SCD genotype beyond what was recorded in diagnosis codes, previous crisis frequency, hydroxyurea use, transfusion history, adherence to prenatal care, blood pressure measurements, or the clinical indications for delivery. Other severe complications associated with hypertensive disorders of pregnancy, including posterior reversible encephalopathy syndrome, were not evaluated as separate outcomes in this analysis.

Because HCUP-NIS captures delivery-related hospitalizations, this analysis was limited to in-hospital complications during the delivery admission and could not evaluate antepartum events, postpartum complications after discharge, or repeat admissions across pregnancy. The unit of analysis was the hospitalization rather than the individual patient, and the NIS does not permit longitudinal linkage of hospitalizations. The cross-sectional design also prevents determination of the temporal sequence between acute crisis and the study outcomes and precludes causal inference.

Although the models were adjusted for maternal age, race and ethnicity, and obesity, the relatively small number of hospitalizations involving acute crisis limited the number of covariates that could be included without overfitting. Type 2 diabetes, chronic kidney disease, and multiple gestation were too uncommon for reliable inclusion in the adjusted models. Residual and unmeasured confounding therefore remain possible. Race and ethnicity and gestational age were each missing for 3.1% of the final cohort, and multivariable analyses used complete cases without imputation. Finally, selection bias is possible because the analytic cohort was restricted to delivery hospitalizations with a recorded diagnosis of SCD and therefore may not capture pregnancies in which SCD or acute crisis was not documented during the delivery admission.

## Conclusions

In conclusion, this national analysis of delivery hospitalizations suggests that acute SCC is strongly associated with severe preeclampsia, eclampsia, or HELLP syndrome among patients with SCD. Acute crisis was also associated with a broader hypertensive disorder composite and cesarean delivery. These findings identify acute crisis as a marker of elevated risk during the delivery hospitalization and support close surveillance, coordinated multidisciplinary care, and timely management of pregnant patients with SCD who experience crisis. Future research should evaluate the temporal relationship between acute crisis and hypertensive disorders, identify modifiable factors associated with crisis during pregnancy, and determine whether strategies that prevent or rapidly control crisis can improve maternal and delivery outcomes.
